# Solid Lipid Nanoparticles: Emerging Colloidal Nano Drug Delivery Systems

**DOI:** 10.3390/pharmaceutics10040191

**Published:** 2018-10-18

**Authors:** Vijay Mishra, Kuldeep K. Bansal, Asit Verma, Nishika Yadav, Sourav Thakur, Kalvatala Sudhakar, Jessica M. Rosenholm

**Affiliations:** 1 School of Pharmaceutical Sciences, Lovely Professional University, Phagwara, Punjab 144411, India; vijaymishra2@gmail.com (V.M.); asitverma234@gmail.com (A.V.); nishikayadav12345@gmail.com (N.Y.); sourav.success.thkr@gmail.com (S.T.); ckbhaipharma@gmail.com (K.S.); 2 Pharmaceutical Sciences Laboratory, Faculty of Science and Engineering, Abo Akademi University, 20520 Turku, Finland; jerosenh@abo.fi

**Keywords:** solid lipid nanoparticles, cytotoxicity, targeted drug delivery, colloidal nanocarriers

## Abstract

Solid lipid nanoparticles (SLNs) are nanocarriers developed as substitute colloidal drug delivery systems parallel to liposomes, lipid emulsions, polymeric nanoparticles, and so forth. Owing to their unique size dependent properties and ability to incorporate drugs, SLNs present an opportunity to build up new therapeutic prototypes for drug delivery and targeting. SLNs hold great potential for attaining the goal of targeted and controlled drug delivery, which currently draws the interest of researchers worldwide. The present review sheds light on different aspects of SLNs including fabrication and characterization techniques, formulation variables, routes of administration, surface modifications, toxicity, and biomedical applications.

## 1. Introduction

**Figure 1:**
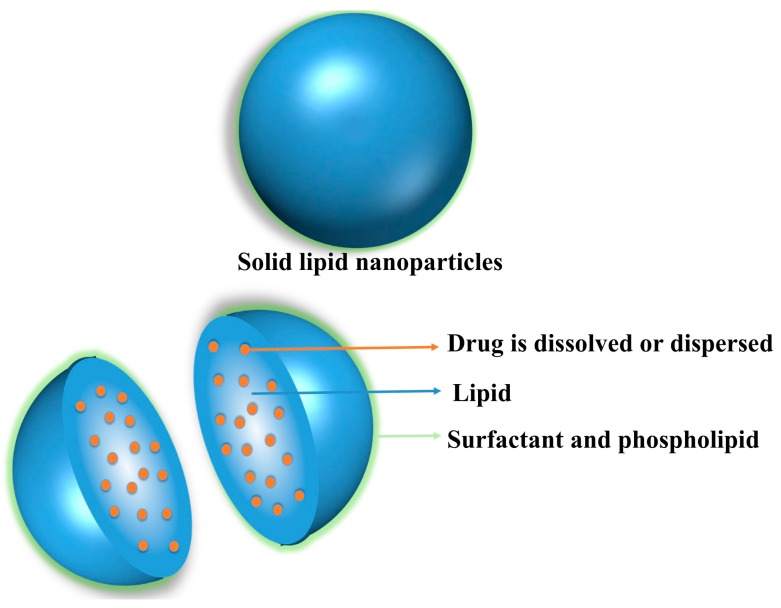
General structure of solid lipid nanoparticle (SLN) loaded with drug.

Solid lipid nanoparticles (SLNs) emerged in 1991 with the objective to provide biocompatibility, storage stability and to prevent the incorporated drug from degradation [1]. SLNs, colloidal carriers of nanoscopic size (50–1000 nm), made up of solid lipids (high melting fat matrix), are developed to conquer the weaknesses (e.g., polymer degradation and cytotoxicity, lack of a suitable large scale production method, inferior stability, drug leakage and fusion, phospholipid degradation, high production cost, and sterilization problems) of traditional colloidal carriers, like polymeric nanoparticles and liposomes [2]. SLNs show various distinctive features such as low toxicity, large surface area, prolonged drug release, superior cellular uptake as compared to traditional colloidal carriers as well as capability to improve solubility and bioavailability of drugs [3,4]. The release of drug from SLNs depends on matrix type and drug location in the formulation. The SLNs fabricated from biodegradable and biocompatible ingredients are able to incorporate both hydrophilic and lipophilic bioactives and thus turning out to be a viable option for controlled and targeted drug delivery [4,5]. The solid core of SLNs is hydrophobic with a monolayer coating of phospholipids and the drug is usually dispersed or dissolved in the core (Figure 1) [5,6,7].

### 1.1. Advantages of SLNs

- The cells of reticuloendothelial system (RES) are unable to take up SLNs because of their nanosize range, thus enabling them to bypass spleen and liver filtration [8,9]
- Provide high stability to incorporated drugs
- Feasibility of incorporating both hydrophilic and lipophilic drugs
- Improve bioavailability of poorly water soluble molecules
- Ease in sterilization and scale up
- Immobilizing drug molecules within solid lipids provides protection from photochemical, oxidative, and chemical degradation of sensitive drugs, with reduced chances of drug leakage
- Drying by lyophilization is achievable
- Provide opportunities for targeted and controlled release of drug
- Biocompatible and biodegradable compositional ingredients [4]

### 1.2. Disadvantages of SLNs

- SLNs are compactly packed lipid matrix networks (ideal crystalline structure) having low space for drug encapsulation, leading to poor drug loading capacity [10,11,12,13]
- Various factors affect the loading or encapsulation of drugs in SLNs, such as interaction of drug and lipid melt, nature or state of lipid matrix, drug miscibility with lipid matrix, and the drug being dispersed or dissolved in the lipid matrix
- Chances of drug expulsion following polymeric transition during storage [14,15]
- The dispersions have a high (70–90%) water content [16]

### 1.3. Nanostructured Lipid Carriers

Nanostructured lipid carriers (NLCs) are developed to conquer the difficulties of SLNs like drug expulsion and low drug loading, as NLCs are prepared from solid and liquid lipid mixture having non-ideal crystalline structure and prevent drug expulsion by avoiding crystallization of lipids [3]. NLCs consist of different spatial lipids (e.g., glycerides) and thus provide a larger distance between the glycerides’ fatty acid chains and general unstructured crystal; and consequently, promote higher drug accommodation. NLCs can be of three different types, viz. imperfect type, multiple type, and amorphous type [16].

- Imperfect type NLCs are prepared by mixing of solid lipids with small amounts of oils (liquid lipids) and thus demonstrate high drug loading.
- In multiple type NLCs, the amount of oily lipids are higher, and therefore yields high drug solubility as compared to solid lipids-. The reason of this phenomenon is based on the fact that the solubility of lipophilic drugs in solid lipids are lower than the liquid lipids (oils).
- Amorphous type NLCs contain additional specific lipids e.g., isopropyl myristate, hydroxyl octacosanyl, hydroxyl stearate etc. to avoid crystallization of solid lipid upon cooling. Thus, expulsion of drug caused by crystallization of solid lipids could be prevented by amorphous type NLCs [16].

NLCs have many advantages like: (a) dispersions of NLC by more solid content can be produced, (b) high capacity of drug-loading as compared to SLNs, (c) modulating drug release profile can be achieved with ease, (d) leakage of drug during storage is less than SLNs, and (e) production of final dosage formulations (e.g., tablets, capsules) is feasible [17].

### 1.4. Lipid Drug Conjugates

Due to partitioning effects, the main problem with SLNs is poor loading of drugs. However, highly potent hydrophilic drugs in low dose can suitably be incorporated in solid lipid matrix. To overcome this difficulty, lipid drug conjugates (LDCs) were utilized, which displayed improvement in drug loading capacities of SLN up to 33%. To produce LDC, first, insoluble drug-lipid conjugate bulk is prepared by salt formation or via covalent linking. Then, it is processed with an aqueous surfactant solution (e.g., Tweens) to prepare nanoparticles by high-pressure homogenization technique. These types of matrices have shown potential in brain targeting of hydrophilic drugs in adverse protozoal infections [18].

## 2. Compositional Profile of SLNs

Lipid and surfactant/stabilizer are the key components used to fabricate SLNs along with co-surfactant, preservatives, cryoprotectant, and charge modifiers (Table 1). By reducing the interfacial tension between the aqueous environment and the hydrophobic surface of the lipid core, surfactants help in stabilizing the SLN structure [4,19].

## 3. Fabrication Techniques of SLNs

Techniques such as High shear homogenization, Ultrasonication or High speed homogenization, Cold homogenization, Hot homogenization, Microemulsion based methods, Supercritical fluid based methods, Solvent emulsification/evaporation methods, Double emulsion methods, and Spray drying methods have been widely employed for the fabrication of SLNs [3,20].

### 3.1. High Shear Homogenization

In this technique, solid lipid nanodispersions are initially produced using high shear homogenization. While handling of the method is easy, the presence of microparticles often compromises the dispersion quality. Investigation of the effect of different process parameters such as stirring rate, cooling condition, and emulsification time on zeta potential and particle size have been investigated. In a study, tripalmitin and mixtures of mono and tri-glycerides (WitepsolW35) were used as lipids with glyceryl behenate (monoester of glycerin and behenic acid) and Pluronic^®^ F-68 as steric stabilizers (0.5% *w*/*w*). Dispersions obtained with WitepsolW35 improved SLN quality by homogenizing at 20,000 rpm for eight minutes. Cooling time kept was 10 min followed by another phase of stirring at 5000 rpm at room temperature [19]. While the polydispersity index (PDI) increased at higher stirring rates, no significant change in particle size was observed.

### 3.2. Ultrasonication or High Speed Homogenization

Ultrasonication or high-speed stirring reduces the shear stress during SLN production. However, some disadvantages are also associated with this method, such as physical instability due to agglomerates or bulky size particles and metal contamination by the high speed of homogenizer in the SLNs formulation [3,4,21].

### 3.3. Hot Homogenization

**Figure 2:**
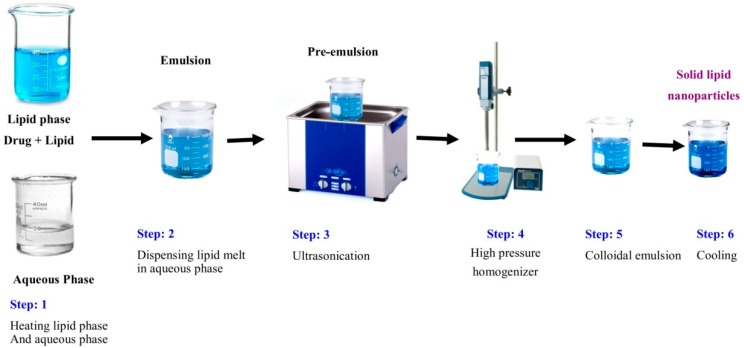
Step by step procedure of hot homogenization technique.

In this method, a pre-emulsion is created by the addition of the lipid melt containing drug and aqueous emulsifier with the help of high shear mixing homogenizer at 500–1500 bar pressure, which reduces the size of the emulsion globules. Minimum five cycles of homogenization is required to get desired size of the globules. The colloidal hot oil in water emulsion is formed after homogenization, which upon cooling causes the crystallization of the lipid in globules and leads to the solid lipid nanoparticles (Figure 2) [22].

### 3.4. Cold Homogenization

Cold homogenization method has been adopted to overcome the problems associated with hot homogenization, such as accelerated drug degradation due to high temperature and loss of drug into the aqueous phase due to partitioning. However, the drug exposure to temperature cannot be eliminated completely in this method, due to drug solubilization in melted lipid and because of the heat generation during the homogenization process. Therefore, the melt containing drug is cooled rapidly using dry ice or liquid nitrogen. This rapid cooling forms the drug solid solution (homogeneous distribution), which is subsequently pulverized to form microparticles by ball/mortar milling. These microparticles are dispersed in chilled aqueous phase containing emulsifier and homogenized subsequently at room temperature for the even allocation of drug in the lipid matrix [4]. Particle sizes attained by this technique are usually in the range of 50–100 nm [23].

### 3.5. Microemulsion Based Method

**Figure 3:**
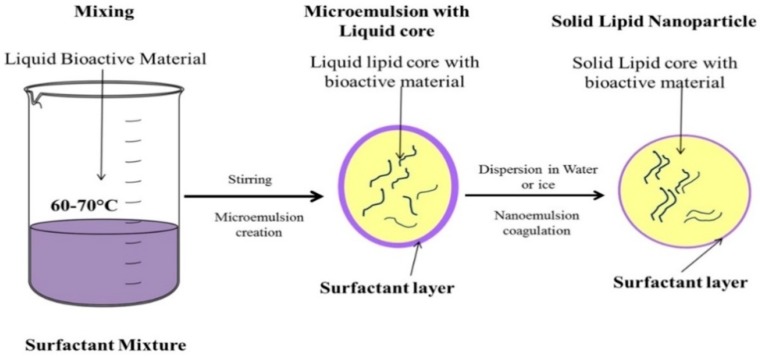
Schematic representation of SLN production by microemulsion technique.

This technique involves dilution of a microemulsion to precipitate the lipid. SLNs are produced by stirring an optically transparent mixture containing a low melting fatty acid, an emulsifier, co-emulsifiers and water at 65–70 °C. After that, the hot microemulsion is dispersed in cold water under stirring. The volume ratios of the hot microemulsion to cold water usually are in the range of 1:25 to 1:50. The dilution process is critically determined by the composition of the microemulsion [19]. This microemulsion is then dispersed in a cold aqueous medium under mild mechanical mixing, which leads to precipitation of the lipid phase in to SLNs. The method is represented in Figure 3.

### 3.6. Supercritical Fluid Based Method

In this method, SLNs are prepared by particles from gas saturated solutions (GSS), thereby providing the advantage of solvent-less processing. SLN can be organized by using the fast expansion of supercritical carbon dioxide solutions [8]. GSS helps in melting the lipid material, whereafter the lipid melt along with GSS will dissolve in the super critical fluid (SCF) under pressure. The saturated solution is sprayed through the nozzle or atomizer, which causes the expansion of solution whereby SCF escapes rapidly leaving behind the fine dry lipid particles. Absence of organic solvents and wide range miscibility of lipids in SCF justify the advantage of this technique [14].

### 3.7. Solvent Emulsification Evaporation Method

**Figure 4:**
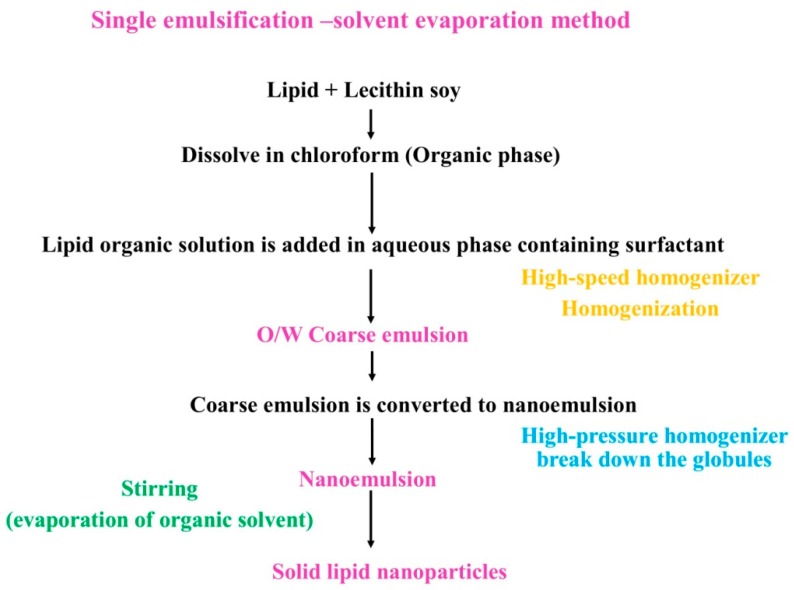
Flow chart for solvent emulsification/evaporation method.

Solvent emulsification evaporation method (SEE) has three basic steps for preparation of nanoparticles. In step (I), lipid material is added to a known volume of organic solvent and mixed properly to yield a homogenous clear solution of lipid. In step (II), above prepared solution is added to the right volume of water in order to form a coarse emulsion by using high-speed homogenizer. Nanoemulsion is then obtained in step (III) by using high-pressure homogenizer, which convert the coarse emulsion into a nanoemulsion due to high pressure, resulting in breakdown of the globules. After nanoemulsion formation, it is kept overnight under continuous stirring on a magnetic stirrer or kept in a hood to remove the traces of organic solvent. Nanodispersion is formed after evaporation of organic solvent, as lipid material will precipitate in the water. The precipitation of lipids in aqueous medium is separated out by filtering through sintered disc filter funnel. Nanoparticles prepared by this strategy are nanosized, non-flocculated (single entity) and have high entrapment efficiency [24,25]. The layout of this method is given Figure 4.

### 3.8. Double Emulsion Method

Double emulsion technique is one of the most frequently used techniques to prepare nanoparticles encapsulated with hydrophilic drugs using stabilizer or surface-active agent [26]. This method is also known as multiple emulsion method, where it has three basic steps: (i) formation of the water in oil emulsion or inverse emulsion, (ii) addition of the W_1_/O emulsion into the aqueous solution of polymer or surfactant to form a W_1_/O/W_2_ emulsion with continuous stirring (sonication or homogenization), and (iii) evaporation of the solvent or filtration of the multiple emulsion to form the nanoparticles. The double emulsion technique produces larger sized particles, than surface modification is achievable through this technique by incorporating hydrophilic polymers such as PEG during step ii [27].

### 3.9. Spray Drying Method

The spray drying method is an alternative procedure to transform an aqueous SLN dispersion into a drug product. This method is barely used for formulation of SLNs; however, it is cheaper than lyophilization. Particle aggregation due to high temperature and shear force, and partial melting of the particles are drawbacks associated with this method [24]. This method requires lipids that have a melting point above 70 °C [28].

## 4. Drying Techniques of SLNs 

### 4.1. Spray Drying

A redispersable powder can be obtained by spray drying, following general requirements of intravenous injections. Addition of carbohydrates and lower amount of lipid during spray drying favor the shielding of the colloidal particles. Lipid melting can be reduced, using ethanol–water mixtures (dispersion medium) rather than pure water due to low inlet temperatures. It was suggested that for the optimum result, SLN concentrations of 1% in solution of 30% trehalose in water or 20% trehalose in ethanol–water mixtures (10/90 *v*/*v*) could be used [29].

### 4.2. Lyophilization

Lyophilization increases the physical and chemical stability of SLN over prolonged storage times. Furthermore, it prevents degradation responses and preserves the initial particle size. SLN ingredients are required for adequate chemical strength and narrow size distribution of particles to circumvent crystal growth. The SLN formulation should be unaffected by temperature variations during shipping. It has been shown that in aqueous SLN dispersions, particle sizes have not undergone alteration over several months. Lyophilization involves surfactant protective effect. However, the lipid content of SLN dispersion should not exceed 5% for avoiding the increase in particle size [29].

## 5. Characterization Techniques of SLNs

Various parameters need to be assessed to understand the fate of SLNs, such as size, particle size distribution, zeta potential, nature and degree of crystallinity, lipid alteration due to polymorphism nature, surface morphology, as well as existence of other colloidal structures (micelles, supercooled melts, and drug nanoparticles) [30].

### 5.1. Particle Size and Zeta Potential

**Figure 5:**
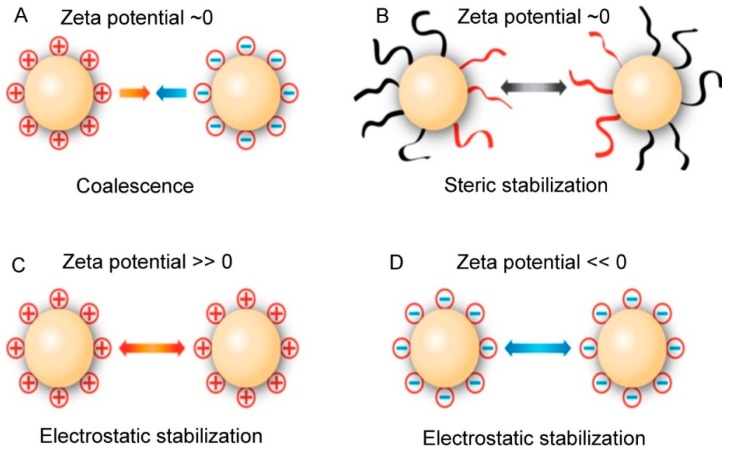
Influence of zeta potential on particle-particle interaction.

Particle size, polydispersity index (PDI) and zeta potential are the essential characteristics of nanoparticles [9]. Dynamic light scattering (DLS) is one of the most important techniques used to characterize SLNs. The speed of analysis, easy sample preparation, and sensitivity to submicrometer particles are the advantages of this method [30]. The size of SLNs is an important factor for their physical stability [31]. Zeta potential measurements can provide information about the colloidal stability of the particles as well as shelf life of colloidal dispersions. As a rule of thumb, high values of zeta potential (e.g., greater than ±30 mV) can stabilize the colloidal dispersion by electrostatic repulsion under given conditions (Figure 5) [30]. Electrostatic repulsion causes the particles to repel each other, thus avoiding aggregation (Figure 5C,D). However, particles with zeta potential near zero under storage conditions may also be stabilized upon storage. Such stabilization can be achieved by coating the particle with a hydrophilic polymer (e.g., PEG) to create a physical barrier against aggregation. This stabilization is known as steric stabilization (Figure 5B). The appropriateness of nanocarrier formulations regarding specific route of drug administration is largely based on the size, size distribution and colloidal stability. Their control and validation are thus of high importance for efficient clinical prospects of nanocarrier preparations [32].

### 5.2. Surface Morphology 

Electron microscopy techniques such as scanning electron microscopy (SEM) gives 3D images of the particles, surface morphology, and transmission electron microscopy (TEM) gives information about the size and shape of nanoparticles as well as internal structure [33].

### 5.3. Degree of Crystallinity 

Degree of crystallinity of lipid particles can be determined with the aid of differential scanning calorimetry (DSC). It is a thermo-analytical technique, which delivers a fast and accurate method for determining the degree of crystallinity of lipids based on the enthalpy of the lipid. Powder X-ray diffractometry (PXRD) is another non-destructive method and widely applied for the description of crystalline materials, and analyze the crystal structure of the SLN [23].

### 5.4. Acoustic Methods

Acoustic spectroscopy is another technique, which measures the attenuation of sound waves as a mean of determining size and surface charge by fitting physically relevant equations. The acoustic energy is applied to the nanoparticles, which introduces charge to the particles because of the movement and generation of the oscillating electric field. Thus generated electric field is utilized to describe the surface charge information [20].

## 6. Scale-Up of SLNs Production

Gasco and co-workers designed an apparatus to fabricate SLNs, which permits dispersion of warm microemulsion in cold water for quick production of larger amounts of SLNs [34]. This apparatus consists of:

- Thermostated aluminum chamber (syringe) containing pneumatically functioned piston for delivering the microemulsion at a designated flux.
- At the bottom of the aluminum chamber, there is a stainless steel support for a sterile membrane filter (0.22 µm), to assure the sterility of the product.
- The stainless steel support is connected with a needle by Lure Lock connection. This apparatus is placed in an electric thermostated jacket. SLNs are formed by dispersing the warm microemulsion into an ice-cooled capsule containing water. The water is stirred by a cylindrical magnetic bar at a fixed rate (300 rpm).
- The microemulsion drops from the needle in the center of the capsule (ice-cooled). The SLN dispersion is stirred for additional 15 min after the widespread microemulsion dripping.

The process factors such as pressure applied to the pneumatic cylinder, needle gauge, temperature of the aluminium chamber, and volume of dispersing water primarily affect the particle size and PDI of SLNs. A temperature difference between warm microemulsion and cold dispersing water plays an important role on the resulting size of the produced SLNs. A rapid crystallization of the oil droplets of the warm microemulsion during quenching favors the formation of small SLNs and avoids coalescence. By the use of a small needle, as well as high pressure and temperature, the SLNs with diameter of about 26 nm, and PDI of 0.1 were obtained [34].

Gohla and Dingler standardized a scaling and production method to manufacture drug free and drug loaded SLNs on medium scale. SLN batches of 2–10 kg were produced by high pressure homogenization technique using a modified Lab 60 device by discontinuous mode. For 50 kg batches, a continuous production mode was used by combining two homogenizers in series. A Gaulin 5.5 device was chosen as first homogenizer to transport the SLN dispersion into the Lab 60 device as a second homogenizer. Homogenization at 500 bar pressure was found to be the ideal pressure condition with 2–3 cycles. The authors demonstrated that production of SLNs can be easily scaled up to industrial scale [3,35].

## 7. Drug Loading and Release Aspects of SLNs

### 7.1. Drug Loading into SLNs

**Figure 6:**
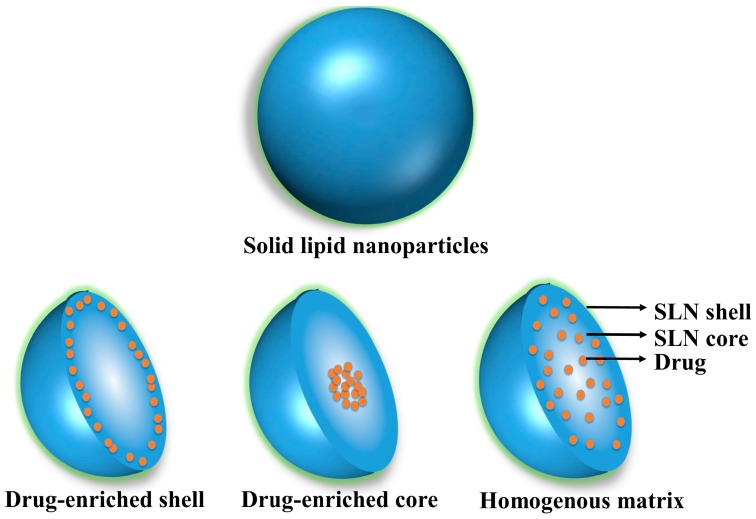
Models of incorporation of active compounds into SLN.

Currently, the fabrication strategy of lipid nanocarriers for controlled and stimuli-responsive drug release has raised research attention for overcoming the problems associated with poorly soluble and toxic drugs. There are mainly three drug incorporation models valid for SLNs: Homogenous matrix model, Drug enriched shell-core shell model, and Drug enriched core-core shell model (Figure 6) [36,37,38].

In homogenous matrix model, the core may consist of drug in either amorphous clusters or molecularly dispersed phase. This model is usually observed when highly lipophilic drugs are incorporated into SLN either by application of hot or cold homogenization method.

In drug enriched shell model, drug is available near the shell, thus yielding a drug free lipid core. A phase separation occurs when the solution is cooled and lipid precipitates out leading to drug free lipid core. During the same period, the drug re-partitions into the remaining liquid-lipid phase and drug gradually increases its concentration in the outer shell of the lipid core.

A drug-enriched core can be formulated by liquefying drug in the lipid to its saturation solubility whereby a nanoemulsion is formed. Supersaturation of the drug in lipid melt occurs during the cooling of the nanoemulsion and causes precipitation of the drug before the precipitation of lipid. Further cooling will lead not only to drug but also lipid precipitation surrounding the drug precipitation, which will act as a membrane towards incorporated drug [39]. 

### 7.2. Drug Release from SLNs

For any formulation, the drug release mechanism is of utmost importance. Drug release from SLNs is attributed by degradation, erosion, or diffusion. The release mechanism of drug from SLN matrix depends on the lipid and its composition. In SLN, drug is either embedded in the matrix or on the surface, and such a system can show versatile release or dual release (immediate release with sustained release). Drug adhered on the surface of SLN will disperse from the nanoparticle and will show an immediate release effect, thereafter the matrix can erode or degrade depending upon the lipid composition, and release the drug in a controlled manner. Temperature or surface-active agents can control drug solubility in water. Temperature based drug release or high amount of surfactant can cause burst release of drug from SLN [40]. Thus, the production of the SLN is usually takes place at room temperature to avoid burst release and partition of drug in aqueous phase. This can lead into partitioning of majority of drug in lipid phase and, therefore, a sustained or controlled release without any immediate release of drug can be observed from SLN. The common ideology of drug release from SLNs depicts that the release is affected by particle size. Smaller particles with large surface area provide rapid drug release as compared to larger particles. Further, drug release also depends on the type of drug entrapment model of SLN, for example, faster drug release can be observed with drug enriched shell model.

Venkateswarlu and Manjunath have performed in vitro release studies on SLN containing clozapine. Release of clozapine followed Weibul and Higuchi equations rather than the first order equation. Drug properties can influence the release via parameters governing the release such as drug solubility (water or lipid soluble), and its interaction with the lipid matrix. Influence of temperature at the time of production can cause solublization of drug in water as the high enthalpy will dissolve the drug, which can lead to deposition of drug on the outer surface of the lipid matrix [41]. 

SLNs have also been tuned to provide drug release in response to external or internal stimuli. Using the concept of solid-liquid transition upon heating, thermoresponsive SLNs have been recently reported. A mixture of lipids (lauric acid and oleic acid, lauric acid, and linoleic acid) was used in this study to fabricate SLNs. Drug release study demonstrated rapid release of loaded 5-fluorouracil (>90%) at 39 °C attributed to the melting of lipid core, whereas 22–34% of drug release was observed at 37 °C due to the solid core [42]. In another study, cholesterol-PEG coated SLNs have been investigated for its pH sensitive drug release pattern. These particles show faster drug release of loaded doxorubicin at pH 4.7 compared to pH 7.4. Depletion of electrostatic attractions between the negatively charged lipid core lauric acid (due to its protonation) and the positively charged doxorubicin was suggested to be responsible for accelerated release at low pH [43].

## 8. Routes of Administration for SLNs

### 8.1. Topical Route

SLNs are commonly used in topical applications due to their biocompatible nature [44]. Lipophilic drug loaded in SLN displayed higher penetration through skin compared to free drug, due to higher exclusivity and hydration of stratum corneum [45]. Upon application, SLNs gradually transform to the stable polymorph and sustained release can be observed. If such polymorphic transitions are controlled by the addition of a surface-active agent, then controlled release of drug from SLNs can be observed [46,47]. 

### 8.2. Pulmonary Route

Pulmonary route has the capacity to deliver drugs in a non-invasive manner with the help of some device or inhaler to reach the systemic circulation, bypassing first pass metabolism or to treat some lung related diseases. Lipid nanoparticle systems are useful in enhancing drug absorption and transport efficacy in alveolar macrophages in the treatment of diseases related to lungs or non-lung diseases [48].

### 8.3. Oral Route

Delivering SLNs through oral route is very easy and can be delivered in suspension form or solid dosage forms such as a tablet, capsule or dry powder. Lopinavir loaded SLNs were developed by Negi et al. to improve the bioavailability of the drug. Lopinavir loaded SLNs were prepared by means of hot self nano-emulsification method. Due to high intestinal lymphatic uptake of drug-SLNs, the lopinavir oral bioavailability was substantially increased [49].

Silva et al. prepared risperidone loaded SLNs for oral delivery and analyzed them for stability, drug release and improvement of bioavailability [50]. Singh et al. developed rifampicin loaded SLN to prevent the hydrolysis of drug in acidic pH. This approach not only prevents the degradation of drug, but also abridges the intimidation of therapy failure [51,52].

### 8.4. Intravenous Administration

Intravenous (*i.v.*) injection is the most studied route of administration for SLNs, particularly for targeted delivery. Yang et al. reported the pharmacokinetics and biodistribution of camptothecin loaded SLN after *i.v.* injection in mice. In comparison to a neat drug solution, SLNs were found to enhance AUC/dose and mean residence times (MRT) especially in brain. The highest accumulation of SLN in brain, compared to free drug among the tested organs, suggested brain targeting potential of this carrier [53].

### 8.5. Ocular Delivery

SLNs showed good permeation property for ocular delivery. The drug release can be sustained or controlled onto the ocular mucosa, which increased the pre-corneal retention time of the drug as compared to conventional ophthalmic solutions [54,55,56,57,58]. Moreover, nanoscopic size of SLN does not cause any blurred vision. However, SLNs aimed for ocular delivery should have to meet specific criteria, like ocular compatibility (Draize rabbit eye test), sterility, isotonicity, and pH value (similar to lachrymal fluid) [59]. 

Khurana et al. employed quality by design (QbD) approach (encouraged by regulatory bodies for improvement of finished product quality) for developing a moxifloxacin ocular nanosuspension. The SLNs prepared by high pressure homogenization technique exhibited sustained release of moxifloxacin from an in-situ gelling system [60].

## 9. Protection of Incorporated Bioactives from Environmental Degradation in SLNs

SLNs contain bioactives inside the core, thus avoid the direct contact of drug with the external environment, and increase the incorporated drug stability. SLNs markedly improve the stability of siRNA, peptides, and proteins by providing protection against proteolytic degradation and may subsequently provide their sustained release [61].

SLNs act as a cage for protecting acid labile drugs from gastric acid degradation. Arteether endoperoxide ring is an antimalarial drug that degrades in gastric acidic medium, which limits it use. However, it was demonstrated that its degradation could be circumvented by incorporating into SLNs, which consequently retained the activity of the drug [62]. SLNs have also been used to stabilize and deliver a DNA vaccine against visceral leishmaniasis [63].

## 10. Surface Modifications of SLNs

Surface engineering of SLNs improves biocompatibility and targetability. Wang et al. developed hyaluronic acid (HA) decorated, Pluronic 85 (P85) coated paclitaxel (PTX) loaded SLN (HA-PTX-P85-SLN) to overcome drug resistance and to increase antitumor efficacy. The SLNs prepared by hot homogenization technique showed a mean diameter of 160 nm and PTX loading content of 4.9%. PTX loaded SLN demonstrated sustained drug release compared to free PTX. This study suggested that HA modified SLN increased tumor accumulation and thus significantly inhibited PTX resistant tumor growth [64].

Recently, Baek et al. modified the SLN surface by coating N-carboxymethyl chitosan (NCC) for enhancing the oral bioavailability of curcumin. The purpose of coating was to reduce the burst release of curcumin from SLN in gastric acidic environment in order to avoid curcumin degradation. In vitro release experiment suggested a negligible amount of drug release in gastric fluid from NCC coated SLN, whereas unmodified SLN exhibit burst release. In contrast, sustained release was observed in simulated intestinal fluid suggesting an advantage of coating to deliver most of the drug to the intestine. Further, higher AUC and C_max_ of curcumin were observed in vivo from NCC modified SLN. These results suggested NCC modified SLN facilitate intestinal absorption by increasing lymphatic uptake (which allows formulations to avoid CYP3A-mediated hepatic first pass metabolism), and by decreasing drug degradation in acidic environment [65].

Similarly, Wang et al. developed chitosan coated cisplatin loaded SLN (CChSLN) for enhanced anticancer activity in cervical cancer. In vitro cytotoxicity assay suggested superior activity of CChSLN towards killing of cancer cells compared to uncoated particles. Higher apoptosis potential of CChSLN compared to uncoated SLN and free drug could be attributed to the enhanced internalization (due to cationic charge) and controlled release of drug obtained from CChSLN formulation [66].

## 11. Applications of Solid Lipid Nanoparticles

**Figure 7:**
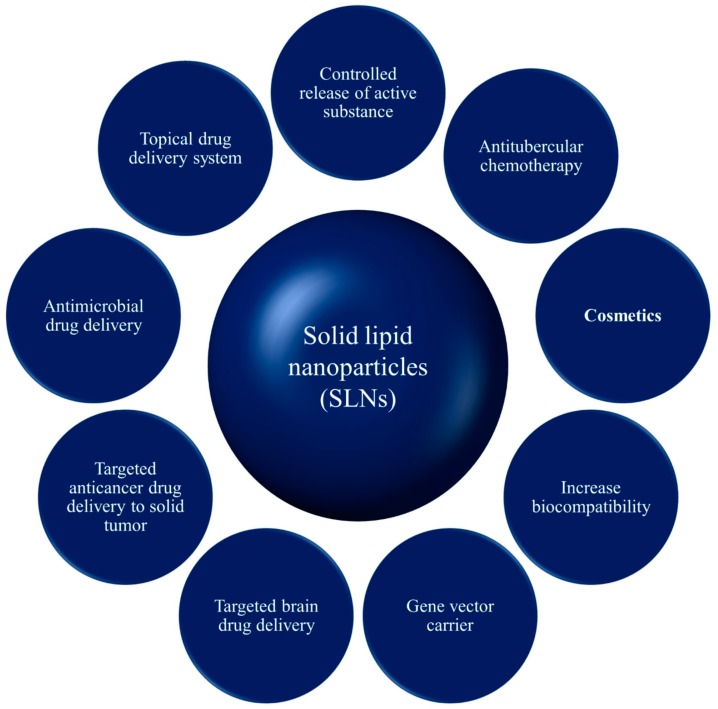
Schematic representation of applications of SLNs.

SLNs enhance the bioavailability of entrapped drugs via modification of the dissolution rate, and can be used to improve tissue distribution and targeting of drugs. Possible applications of SLNs are represented in Figure 7.

### 11.1. Controlled Release of Drug

SLNs offer an advantage to modulate release of loaded drug either by varying drug loading approach or by altering surface properties or composition. In a recent study, SLN loaded with TNF-α siRNA was developed to achieve its prolonged release in treatment of rheumatoid arthritis. SLNs were prepared via a solvent displacement method using biocompatible lecithin and cholesterol, and a complex of siRNA with 1,2-dioleoyl-3-trimethylammonium-propane was encapsulated therein. In vitro release study of siRNA from SLNs demonstrates absence of burst release, and only 5% of siRNA was released in 30 days. This prolonged release property without burst release was attributed to the presence of cholesterol and complex of siRNA in formulation [67]. 

Cavalli et al. prepared inclusion complexes of hydrocortisone and progesterone with cyclodextrin by co-precipitation method. Inclusion complexes were later incorporated into different types of SLNs. The authors observed a delayed release of drug from SLNs of drug-cyclodextrin complex [68]. Achieving controlled release of hydrophilic drugs using SLNs as a carrier is usually challenging due to poor drug loading. However, controlled release of a hydrophilic peptide drug, gonadorelin, was achieved using SLN due to the ability of this carrier to load high amount of gonadorelin (up to 69.4%) by solvent diffusion technique. Drug release behavior from this monostearin SLNs exhibited a biphasic pattern. After burst release (24.4% during first 6 h), a distinctly prolonged release for over 12 days was observed [69].

Jain et al. formulated an anti-acne SLN-based hydrogel for topical delivery of adapalene for treatment of acne [70]. Kim et al. prepared a novel formulation based on a pH-sensitive system. In this system, curcumin loaded SLNs, which act as depot, were coated with mesoporous silica matrix, to control the release of curcumin. A pH dependent release was observed from this complex, which could be attributed to the interaction between silanols of the mesopore surface and curcumin [71].

### 11.2. SLNs for Targeted Brain Drug Delivery

SLNs can improve the ability of the drug to penetrate through the blood-brain barrier (BBB). Abbas et al. targeted clonazepam to brain via intranasal olfactory mucosa utilizing nanolipid carriers that were co-loaded with superparamagnetic iron oxide nanoparticles (SPIONs), both for the guidance of nanocarrier and holding in external magnetic field. The nanolipid carriers are incorporated in situ in thermosensitive mucoadhesive gels, resulting in the enhanced delivery of clonazepam. This study raises the light on new intranasal management of epilepsy with reduction in clonazepam peripheral harmful effects [72].

### 11.3. SLNs for Anticancer Drug Delivery

Recently, SLNs bearing anti-neoplastic drug have been investigated for breast cancer treatment, and results showed a sustained release of tamoxifen with good therapeutic activity [73]. Surface modified SLNs can be fabricated for tumor targeting purposes with help of a suitable targeting ligand for effective delivery of some anticancer drugs like methotrexate (MTX) and camptothecin [74].

Gomes et al. developed lipid core nanoparticles (LDE) containing antiproliferative agent PTX and reported the reduction in atherosclerosis lesions induced in rabbits through cholesterol feeding. After withdrawal of feeding of cholesterol, as compared to LDE-single group, the LDE-PTX and LDE-PTX+LDE-MTX managements has the ability to rise by 49 and 59% plaque areas regression, respectively. The tumor necrosis gene expression factor α was decreased by 65 and 79% using LDE-PTX and LDE-PTX+LDE-MTX, respectively. This result showed the action of combined chemotherapy for achieving higher effects on strongly atherosclerotic inflamed lesions [7].

Chirio et al. developed distearoyl-floxuridine loaded SLN having 70.8–82.8% entrapment ability. In vitro cytotoxicity study performed on human cancer cell lines like HT-29, MDA-MB231 and M14 cells suggested the superior activity of distearoyl-floxuridine SLN towards cancer cell killing. The distearoyl floxuridine SLNs were found to be 100 times more efficient as compared to free floxuridine. Furthermore, clonogenic assay suggested higher cytotoxicity of distearoyl-floxuridine SLN as compared to free drug [75].

### 11.4. SLNs for Antimicrobial Drug Delivery

SLNs release antimicrobial payloads for the effective elimination of infectious microbes harbored at lymphatic sites [8]. Nanoparticles and the nanostructured surfaces oppose the growth of bacteria and infections, which is an effective solution regarding difficulties related to biofilm and antibiotic resistance. SLNs are manufactured for delivery of antimicrobial agents and act against microbes by encapsulating the antimicrobial drugs, disruption of microbial adherence, and receptor-based binding to cellular surfaces [76].

### 11.5. SLNs as Gene Carrier

Several studies have been carried out on SLN bearing genetic materials such as plasmid deoxyribonucleic acid (p-DNA), DNA, and other nucleic acids [7]. Vicente-Pascual et al. reported that SLN based vectors could act as a beneficial system of gene delivery for management of corneal diseases and inflammation [77].

### 11.6. SLNs for Topical Use

SLNs are used topically to deliver various drugs such as vitamin A, sisotretinoin, and flurbiprofen. The flurbiprofen-loaded SLN gel can be applied directly to the site of action, to induce higher tissue concentrations of the drug in controlled fashion [20].

SLN loaded diflunisal (DIF), a non-steroidal anti-inflammatory drug, has also been developed for effective management of rheumatoid arthritis. SLNs formulated by hot homogenisation method (based on microemulsification technique) were spherical in shape with a mean size of 124.0 ± 2.07 nm (PDI 0.294 ± 0.15). These SLNs showed significant decrease in fluid volume, granuloma tissue weight, leukocyte count/mm^3^ in mice air pouch model. Similarly, in mice ear oedema and rat paw oedema model, 2.30 and 1.29 times increase in percentage inhibition of oedema was observed respectively, using SLN formulation compared to conventional cream [78]. 

### 11.7. SLN in Cosmetics

SLNs are novel nanocarriers that can replace the conventional delivery systems such as creams, gels, ointments usage in cosmetics [29,79]. Gonçalez et al. found that curcumin (CUR) have therapeutic properties against skin disorders (SD). The cationic SLNs (CSLN) loaded with CUR were developed and analyzed physicochemically for SD. It was suggested that the surface charge of CSLN (zeta potential, +23.1 to +30.1 mV) played a major role in selective accumulation of drug to the diseased tissue [80].

Jose and Netto compared lipid nano-based systems and traditional cosmetic products on account of occlusiveness. The film formed via lipid nanoparticles on skin was smooth in comparison to film formed using a traditional paraffin product. SLNs based products showed great activity of UV-blocking and photoprotection [29].

### 11.8. SLNs as Adjuvant for Vaccines

Immunologic adjuvants are substances that are used to augment the degree, stimulation, or robustness of vaccines. In this sequence, Stelzner et al. developed squalene containing steam sterilized SLNs based adjuvant system for a yeast-based vaccine. Size of squalene loaded SLN measured by static and DLS technique was found to be in the range of 120–170 nm. Evaluation of the developed vaccine adjuvant on a mouse model showed excellent efficacy against the harmful bursal virus disease. Squalene-based adjuvants represented high biocompatibility and also demonstrated immune stimulation properties, which is comparable with Freund’s adjuvant [81].

### 11.9. SLNs in Antitubercular Chemotherapy

In the current scenario, SLNs and other nanocarriers are utilized to eradicate *Mycobacterium tuberculosis* completely [82]. Castellani et al. reported that SLNs could work as efficient drug delivery system for the natural anti-oxidants derived from seed of grape in oxidative stress model in airway epithelial cells. The authors pointed long-term persistence and stability inside cells and liberation of proanthocyanidins. Their results create a path for novel anti-inflammatory and anti-oxidant therapies for chronic respiratory diseases [83].

### 11.10. SLNs in Bioimaging

The detection and removal of lipopolysaccharides (LPS) from pharmaceutical preparations and food is vital for safe administration and to prevent septic shock. An abiotic system prepared using SLNs aim at reversible capture, detection, and removal of LPS in aqueous solutions. Furthermore, the regenerated particles also act as colorimetric labels in the dot blot bioassays for basic and prompt evaluation of the LPS elimination [84].

In the advanced field of nanomedicine, diverse approaches for rheumatoid arthritis (RA) therapy are available. Albuquerque et al. developed anti-CD64 antibody anchored SLN based theranostic system consisting of SPIONs and MTX (co-encapsulated in the SLNs) for targeting the macrophages in RA. The formulations have sizes lower than 250 nm and -16 mV zeta potential with suitable features for intravenous administration. TEM photographs showed that SPIONs were encapsulated within SLN matrix and obtained values of MTX association efficiency were greater than 98%. In vitro studies demonstrated that all formulations exhibited low cytotoxicity up to 500 μg/mL concentration in THP-1 cells. The SLN based formulations are, therefore, promising candidates for both therapeutic and imaging purposes [85].

## 12. Toxicity Aspects of SLNs

Materials used in drug delivery systems should be biocompatible and assessment of biocompatibility is an obligatory viewpoint to address. While an exact assurance of the toxicity of a formulation must be resolved through in vivo studies, an assortment of In vitro toxicological assays, performed in satisfactorily selected cell lines, may give extremely helpful data. These tests are broadly acknowledged as first markers of toxicity [86].

### 12.1. Cytotoxicity of SLNs

Assurance of cell toxicity or cell viability remains the furthermost regular test utilized as confirmation of biocompatibility or toxicity. SLN prepared using glyceryl monostearate has been tested for their cytotoxicity In vitro on monkey kidney epithelial cells (VERO) and acute lymphoblastic leukemia cells (L1210) using MTT assay. The 50% inhibitory concentration (IC_50_) of SLN was found to be 0.7 and 0.4 mg/mL in VERO cells and 0.5 and 0.3 mg/mL in L1210 cells, after 24 and 48 h of incubation, respectively [87].

In another study SLNs prepared using Softisan^®^ 154 and soy lecithin via high-pressure homogenization technique were tested on MCF-7 and MDA-MB231 for their toxicity. The IC_50_ values reported in this study for MCF-7 cells were found to be approximately 0.28, 0.26, 0.22 mg/mL after 24, 48 and 72 h, respectively. Similarly, IC_50_ values observed for MDAMB-231 cells were found to be about 0.29, 0.29, 0.27 mg/mL after 24, 48, and 72 h, respectively [88]. It can be concluded that the lipid used to prepare nanoparticle has significant effect on the cytotoxicity of obtained SLNs.

#### 12.1.1. Impact of Surface Charge

The interaction between the colloidal nanoparticles and cells depend on the surface charge of the particles. Cationic surfactants used in SLNs can create deformities in membrane integrity [89] and sensitize the immune system [90].

#### 12.1.2. Effect of Composition on Cell Viability

Identification of the surfactants used for SLNs, not only in terms of biocompatibility but also for the stability or shelf life, is something very important for the SLNs system. Pluronic^®^ F-68 and Tween 80 were used in topical, oral liquid, and semisolid dosage forms. Assessment of both surfactants (Pluronic^®^ F-68 and Tween 80) for cell viability incorporated in SLNs was made. Pluronic^®^ F-68 in SLNs has shown good stability and 90% cell viability, whereas Tween 80 in SLNs with same lipid composition has shown better stability but with 50% cell viability [91]. The nature of surfactant used in SLNs and duration of contact time of SLNs with cells will influences the cell viability percentage [92].

### 12.2. Genotoxicity

Several studies suggested that SLN does not show any damage to DNA or gene related toxicity. Dolatabadi et al. and Bhushan et al. investigated SLN with negative charge by incubating with A549 cells, and found that these did not produce any toxicity or harm to genome DNA determined by gel electrophoresis [92,93]. However, a report suggested damage in DNA by acetyl shikonin-bearing SLN, which instigated an increase in comet development in A549 cells. SLN-encapsulated drug further increased the DNA damage [94].

### 12.3. Hemolytic Toxicity

Hemolysis examination was usually performed to evaluate the extent of red blood cell destruction caused by *i.v.* injection of foreign material [95]. Lakkadwala et al. evaluated SLNs consisting of glycerol monostearate and polysorbate 80 for their hemotoxicity, and the obtained results demonstrated low hemotoxicity of SLN even at high dose (1 mg/mL) [73]. Hyaluronic acid coated SLN bearing antineoplastic drug also demonstrated low hemolytic toxicity, regardless of whether the formulation displayed a cationic surface or an anioinic surface [96]. Another cationic SLN bearing doxorubicin was found to be non-hemolytic. This impact was additionally articulated when SLNs were covered with galactose [97].

## 13. Marketed Formulations of Solid Lipid Nanoparticles

To enhance the bioavailability of BCS class II drugs, lipid-based formulations have been utilized. Around 4% of commercially available products in the United States, United Kingdom, and Japan market are oral lipid based formulations. Oral lipid based systems vary from simple lipid solutions to self-emulsifying drug delivery systems (SEDDS) [98].

## 14. Conclusions and Future Perspectives

SLNs are an amalgamation of the properties of liposomes and polymer based carriers, where encapsulation of both lipid soluble and water soluble drugs could be possible. Production of SLN is inexpensive, and scale up is feasible. They pose high stability during their shelf life, and a wide range of lipids are available for tuning the release kinetics. SLNs have emerged as efficient drug delivery systems and the future of lipid based drug delivery is largely dependent on SLNs due to their various significant properties. Scientists have already filed many patents related to SLNs and we can anticipate more patented SLN-based delivery systems in the near future.

## Figures and Tables

**Table 1:** Ingredients used in SLNs-based formulations.

Ingredients	Examples
Lipid component	Beeswax, Stearic acid, Cholesterol, Caprylic/capric triglyceride, Cetylpalmitate, Glyceryl stearate (-mono, and -tri), Glyceryl trilaurate, Glyceryl trimyristate, Glyceryl behenate (Compritol), Glyceryl tripalmitate, Hardened fat (Witepsol E85, H5 and W35), Monostearate monocitrate, Solid paraffin, Behenic acid
Surfactant/Emulsifiers	Phosphatidyl choline, Soy and Egg lecithin, Poloxamer, Poloxamine, Polysorbate 80
Co-surfactant	Sodium dodecyl sulphate, Tyloxopol, Sodium oleate, Taurocholate sodium salt, Sodium glycocholate, Butanol
Preservative	Thiomersal
Cryoprotectant	Gelatin, Glucose, Mannose, Maltose, Lactose, Sorbitol, Mannitol, Glycine, Polyvinyl alcohol, Polyvinyl pyrrolidone
Charge modifiers	Dipalmitoyl phosphatidyl choline, Stearylamine, Dicetylphosphate, Dimyristoyl phophatidyl glycerol
